# Subsoiling-Induced Shifts in Nitrogen Dynamics and Microbial Community Structure in Semi-Arid Rainfed Maize Agroecosystems

**DOI:** 10.3390/microorganisms13081897

**Published:** 2025-08-14

**Authors:** Jian Gu, Hao Sun, Xu Zhou, Yongqi Liu, Mingwei Zhou, Ningning Ma, Guanghua Yin, Shijun Sun

**Affiliations:** 1 Institute of Applied Ecology, Chinese Academy of Sciences, Shenyang 110016, China; gujian@iae.ac.cn (J.G.); haos@spaces.ac.cn (H.S.); zhouxu20@mails.ucas.ac.cn (X.Z.); 2022200011@stu.syau.edu.cn (Y.L.); zhoumingwei22@mails.ucas.ac.cn (M.Z.); ma_ningning23@163.com (N.M.); 2 University of Chinese Academy of Sciences, Beijing 100049, China; 3 College of Water Conservancy, Shenyang Agricultural University, Shenyang 110866, China

**Keywords:** subsoiling, nitrogen cycling, microbial community

## Abstract

Global agricultural intensification has exacerbated soil compaction and nitrogen (N) inefficiency, thereby threatening sustainable crop production. Sub-soiling, a tillage technique that fractures subsurface layers while preserving surface structure, offers potential solutions by modifying soil physical properties and enhancing microbial-mediated N cycling. This study investigated the effects of subsoiling depth (0, 20, and 40 cm) on soil microbial communities and N transformations in a semi-arid maize system in China. The results demonstrated that subsoiling to a depth of 40 cm (D2) significantly enhanced the retention of nitrate-N and ammonium-N, which correlated with improved soil porosity and microbial activity. High-throughput 16S rDNA sequencing revealed subsoiling depth-driven reorganization of microbial communities, with D2 increasing the abundance of Proteobacteria (+11%) and ammonia-oxidizing archaea (Nitrososphaeraceae, +19.9%) while suppressing denitrifiers (nosZ gene: −41.4%). Co-occurrence networks indicated greater complexity in microbial interactions under subsoiling, driven by altered aeration and carbon redistribution. Functional gene analysis highlighted a shift from denitrification to nitrification-mineralization coupling, with D2 boosting maize yield by 9.8%. These findings elucidate how subsoiling depth modulates microbiome assembly to enhance N retention, providing a mechanistic basis for optimizing tillage practices in semi-arid agroecosystems.

## 1. Introduction

The acceleration of global agricultural intensification has rendered soil physical degradation and nutrient use inefficiency critical constraints to sustainable agricultural development. According to statistics from the Food and Agriculture Organization of the United Nations (FAO), approximately 160 million hectares of global cropland are affected by soil compaction, resulting in a 30–50% increase in root penetration resistance and a 20–35% reduction in nitrogen partial factor productivity (FAO, http://www.fao.org/publications, accessed on 6 June 2025). Concurrently, the excessive application of chemical nitrogen fertilizers, which exceeds 110 million tons annually, leads to a nitrogen loss of 45–70% and contributes to environmental issues, including groundwater nitrate contamination (greater than 50 mg/L) and a 1.3% annual increase in N_2_O emissions (IPCC, https://www.ipcc-nggip.iges.or.jp/efdb/find_ef.php, accessed on 6 June 2025). In this context, subsoiling has emerged as an innovative tillage technique that simultaneously addresses soil improvement and nitrogen cycle regulation; however, its underlying mechanisms require systematic elucidation [1].

Subsoiling is a specialized tillage practice that fractures subsurface soil layers at depths of 20 to 50 cm while preserving the structure of the surface soil. This technique employs implements such as subsoiler shanks or vibrating tines [2]. Compared to conventional tillage, its key advantages include effectively breaking up the plow pan, reducing soil bulk density from 1.6–1.8 g/cm^3^ to 1.3–1.4 g/cm^3^ [3], and establishing vertically connected pore networks (with a 15–20% increase in macropores > 100 μm diameter) that significantly improve soil phase composition [4]. These physical modifications create a unique microenvironment that enhances microbial-mediated nitrogen cycling processes [5].

Emerging evidence reveals that subsoiling regulates nitrogen cycling through a “physical–biological” coupling mechanism. Physically, the created macropores enhance gas diffusion (increasing O_2_ diffusion coefficient by 2.1-fold) and boost potential nitrification rates by 40–60% [6]. Biologically, it restructures nitrogen-transforming microbial communities via three mechanisms as follows: (1) three-dimensional pore architecture expands the niche width of ammonia-oxidizing archaea (AOA) by 1.8-fold [7], significantly increasing *amoA* gene copies [8]; (2) deep straw incorporation (30 cm) creates anaerobic microsites for nitrogen-fixing bacteria (e.g., *Bradyrhizobium*), elevating *nifH* gene abundance by 2–3 orders of magnitude [9]; and (3) increased fungal-to-bacterial ratio facilitates nitrogen translocation through fungal hyphal networks [10]. This microbial restructuring drives fundamental changes in nitrogen cycling: (1) accelerated mineralization-immobilization turnover (MIT) increases amino sugar-bound nitrogen; (2) downward shift of nitrification hotspots reduces NH_4_^+^ leaching risks in surface soils [11]; and (3) suppressed electron transport chain activity in denitrifiers (e.g., *Pseudomonas*) decreases N_2_O emission factors by 0.2–0.4 percentage points [12].

Western Liaoning, as a representative semi-arid region, exhibits globally significant challenges in nitrogen management within its agricultural ecosystems [13]. Characterized by scarce and unevenly distributed precipitation (annual mean 400–500 mm), this region suffers from severe nitrogen loss through leaching and runoff, while concurrently facing dual constraints of insufficient nitrogen supply and imbalanced nitrogen forms (e.g., excessive nitrate proportion) on crop productivity [14]. Studies indicate that approximately 60% of cultivated lands in this region demonstrate nitrogen use efficiency below 40%, with traditional tillage exacerbating mineralization-immobilization imbalance, further reducing amino sugar-bound nitrogen accumulation by 15–20% [3]. As a core conservation agriculture technology, subsoiling has been proven to significantly improve soil water retention capacity and nitrogen conservation efficiency in semi-arid regions [15]. However, its fundamental mechanism in regulating nitrogen cycling—specifically the process of shaping divergent microbial community structures through varying tillage depths and consequently influencing nitrogen transformation pathways—remains systematically unquantified.

Emerging evidence reveals depth-dependent microbial restructuring: Shallow subsoiling at 30 cm increases ammonia-oxidizing archaea (AOA) *amoA* gene abundance by 1.5-fold, but concurrently elevates denitrifier *narG* gene abundance by 40%, potentially raising N_2_O emission risks [16]. In contrast, deep subsoiling at 50 cm enhances diazotroph *nifH* gene copies by two orders of magnitude, yet may suppress nitrification due to restricted oxygen diffusion [15]. This depth-specific microbiome remodeling directly governs critical nitrogen transformation processes: shallow subsoiling accelerates mineralization-immobilization turnover (35% higher MIT rate) but intensifies surface nitrogen volatilization; deep subsoiling promotes nitrate migration to subsoil (20–30 cm downward shift) at the potential cost of reduced short-term plant availability.

Critical knowledge gaps persist: (1) Quantitative models describing variations in the abundance and taxonomic profile of key soil functional genes (e.g., *amoA*, *nirK*, *nifH*) across subsoiling depth gradients (30, 40, and 50 cm) remain lacking. (2) The cascade effects of pore structure-microbe interactions on nitrogen speciation, particularly dynamic anaerobic/aerobic microzone formation in 20–50 cm soil layers, remain unelucidated [17].

This study investigated the effects of subsoiling depth (0, 20, and 40 cm) on soil microbial communities and nitrogen cycling in a semi-arid maize cropping system (Fuxin County, China). A randomized block design (*n* = 12 plots) was implemented, with soils sampled during the 2020 growing season for physicochemical (TN, DOC, NH_4_^+^/NO_3_^−^) and high-throughput molecular analyses (16S rRNA sequencing, qPCR of N-cycle genes). The research aimed to (1) quantify depth-dependent shifts in microbial α/β-diversity and co-occurrence networks, (2) investigate Tillage practice impacts on soil structure and associated functional gene abundance shifts, and (3) elucidate mechanistic links between microbial restructuring and N transformation processes. Statistical integration of biogeochemical and omics data provides novel insights into how subsoiling depth modulates microbiome assembly to enhance N retention in water-limited agroecosystems.

## 2. Materials and Methods

### 2.1. Experimental Site Description

**Figure 1:**
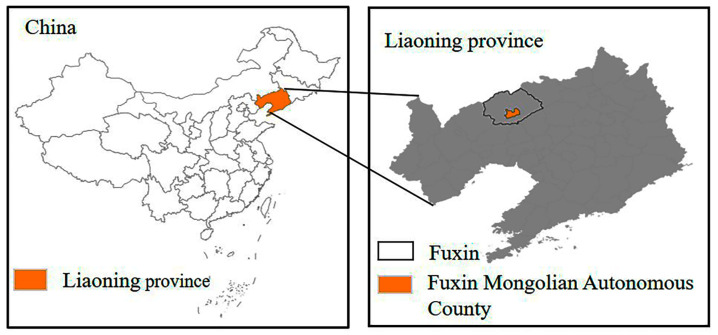
A map of the study area and its overview.

The study was conducted in Fuxin Mongolian Autonomous County, Liaoning Province (41°41′–42°56′ N, 121°01′–122°56′ E; mean elevation: 235 m) (Figure 1), characterized by a temperate semi-arid continental monsoon climate. Meteorological data (2015–2020) indicated an annual mean temperature of 7.1–7.6 °C and precipitation of 493.1 mm (±62.3 mm SD), with 68% (335.3 ± 48.7 mm) occurring from June to August. This study was conducted in 2023, and the precipitation and mean temperature during the experimental period are presented in Table S9.

Annual evaporation averaged 1847.6 mm (±213.5 mm). The soil was classified as Haplic Luvisols (FAO) with a sandy loam texture (62.3% sand, 24.1% silt, 13.6% clay). The topsoil (0–20 cm) had a bulk density of 1.42 ± 0.08 g·cm^−3^, field capacity of 23.00% ± 1.25%, and pH of 6.15 ± 0.23 (soil/water = 2.5:1). Soil nutrient contents included total nitrogen (0.76 ± 0.05 g·kg^−1^, Kjeldahl method), alkali-hydrolyzable nitrogen (119.50 ± 9.32 mg·kg^−1^), available phosphorus (8.12 ± 0.67 mg·kg^−1^, Olsen method), total potassium (17.73 ± 1.24 g·kg^−1^, flame photometry), available potassium (104.66 ± 8.53 mg·kg^−1^, NH_4_OAc extraction), and organic matter (15.67 ± 1.02 g·kg^−1^, K_2_Cr_2_O_7_ oxidation). Primary crops were maize (*Zea mays* L.).

### 2.2. Experimental Design

A completely randomized block design was employed, with plots arranged randomly (using a random number table). The main treatment factor was subsoiling depth (D-Depth), with three levels: no-tillage without subsoiling (D0, flat tillage), no-tillage with 20 cm subsoiling (D1, ±1.5 cm), and no-tillage with 40 cm subsoiling (D2, ±2.1 cm; performed using a hydraulic subsoiler). Conventional tillage (straw removal + rotary tillage at 10–15 cm depth; CK) served as the control. The experiment comprised four treatments with three replicates each (*n* = 12 plots). Each plot measured 6 m × 8 m (48 m^2^, long edge parallel to prevailing winds). Subsoiling was conducted at the maize V3 stage (20 June, within 24 h after rainfall). To minimize disturbance to the root system, the subsoiler was operated exclusively between the rows.

### 2.3. Soil Sampling and Chemical Analysis

The sampling was conducted on 10 May (sowing), 30 June (jointing), and 25 September (harvesting), 2023, for corn fields.

Soil samples were collected in rows from each plot (0–20 cm depth) using a 2 cm inner diameter stainless steel corer (Eijkelkamp, Giesbeek, The Netherlands) following an S-shaped sampling pattern (5-point method) [18]. A total of six soil cores (diameter × height = 2 cm × 20 cm) per plot were homogenized by quartering to form a composite sample (~500 g fresh weight). During 15–20 September 2020 (before crop harvest), each fresh composite sample was divided into three portions: (1) 50 g was immediately flash-frozen in liquid nitrogen and stored at −80 °C (Thermo Scientific, Waltham, MA, USA) for subsequent DNA extraction (storage duration < 3 months); (2) 100 g was refrigerated at 4 °C (<24 h) for analyses of microbial biomass (chloroform fumigation-K_2_SO_4_ extraction), dissolved organic carbon (DOC; 0.5 M K_2_SO_4_ extraction), and dissolved organic nitrogen (DON; potassium persulfate oxidation); and (3) the remaining sample was air-dried in the dark at 25 ± 3 °C for 7 days, sieved through a 2 mm mesh, and stored in a desiccator for physicochemical analyses. DOC was extracted from soil slurries (1:5 soil/water ratio), followed by filtration through Whatman GF/F membranes [19]. The filtrates were stored at −18 °C in 50 mL centrifuge tubes (light-protected) before analysis. After thawing at 25 °C for 2 h, DOC concentrations were determined using a high-temperature combustion total organic carbon analyzer (TOC-VCPH, Shimadzu, Kyoto, Japan; detection limit: 0.1 mg/L). NH_4_^+^-N (indophenol blue method) and NO_3_^−^-N (UV spectrophotometry) were extracted with 2 mol/L KCl (1:5 soil/solution ratio) and quantified using a flow injection analyzer (Skalar SAN++, Skalar, Breda, The Netherlands) [20].

### 2.4. DNA Extraction Illumina Sequencing

Samples for analyzing the soil microbial community structure (16S) were collected during the harvest period. Total DNA was extracted from 0.5 ± 0.02 g of soil using the FastDNA SPIN Kit (MP Biomedicals; triplicates, Solon, OH, USA). Concentration (NanoDrop 2000, Thermo Fisher Scientific, Waltham, MA, USA) and purity (A_260_/_280_ = 1.8–2.0) were verified, with integrity confirmed by 1% agarose gel electrophoresis (>10 kb bands). The V3–V4 region of 16S rRNA genes (~468 bp) was amplified with primers 338F/806R using Phusion^®^ High-Fidelity PCR Master Mix(TIANGEN Biotech, Beijing, China) (98 °C 30 s; 25 cycles: 98 °C 10 s, 55 °C 30 s, 72 °C 30 s; 72 °C 5 min). Amplicons were sequenced on Illumina MiSeq (2 × 300 bp; Majorbio, Shanghai, China). Reads were processed in QIIME2 (DADA2; maxEE = 2, truncLen = 250) to generate ASVs (100% similarity). Taxonomy was assigned via SILVA 138 (99% confidence threshold: 0.7).

### 2.5. Functional Gene Quantification

Genomic DNA (0.5 ± 0.01 g soil, triplicates) was extracted (FastDNA Kit) and quantified (Qubit™ 3.0, Thermo Fisher Scientific, Waltham, MA, USA; dsDNA BR Assay). Eight N-cycle genes (*nifH*, *amoA-AOA/AOB*, *nirK*, *nirS*, *nosZ*) were amplified via SmartChip qPCR (Wafergen, Fremont, CA, USA; 384-well chip) using validated primers (55–60 °C annealing). Reactions included non-template controls (NTCs) and standards (R^2^ > 0.99, efficiency: 90–110%) [21]. All primer sets were literature-derived and rigorously validated for target specificity and PCR amplification efficiency [22].

### 2.6. Statistical Analysis

All statistical analyses were conducted using SPSS v25.0 (IBM Corp., Armonk, NY, USA) and R software (v4.3.1) with the microeco package [23] (v2.5–7). Microbial α-diversity was assessed through four complementary indices: observed species richness (Sobs), Chao1 estimator, Shannon diversity index, and phylogenetic diversity (PD) calculated using the vegan::diversity function. Treatment effects on these diversity metrics were evaluated through one-way analysis of variance (ANOVA) with post hoc Duncan’s multiple range tests (significance threshold α = 0.05).

For β-diversity analysis, community composition was visualized via principal coordinates analysis (PCoA) based on Bray–Curtis dissimilarity matrices [24], with significant differences between treatment groups confirmed by analysis of similarities (ANOSIM; R > 0.3 considered biologically meaningful). Pairwise associations between soil variables and microbial taxa were identified through Pearson correlation analysis with false discovery rate (FDR) correction (|r| > 0.6, *p* < 0.05 considered strong relationships). Indicator species analysis was conducted using Threshold Indicator Taxa Analysis with 500 bootstrap iterations and stringent purity thresholds (>0.95).

Co-occurrence network analysis was performed on operational taxonomic units (OTUs) with relative abundance exceeding 0.01%, following the Hellinger transformation. Robust associations were identified through Spearman’s rank correlation. Network visualization and topological analysis were implemented in Gephi v0.9.3 using a Fruchterman–Reingold force-directed layout. Key network properties, including node count, edge number, average degree, characteristic path length, network diameter, clustering coefficient, graph density, and modularity (Louvain algorithm), were computed using igraph (v0.10.15) software. Ecologically important hub taxa were identified through dual criteria, within-module connectivity (Zi > 2.5) and among-module connectivity (Pi > 0.62), representing module hubs and network connectors, respectively.

## 3. Results

### 3.1. Effects of Subsoiling on Soil Nitrate-N (NO_3_^−^-N) Dynamics

**Figure 2:**
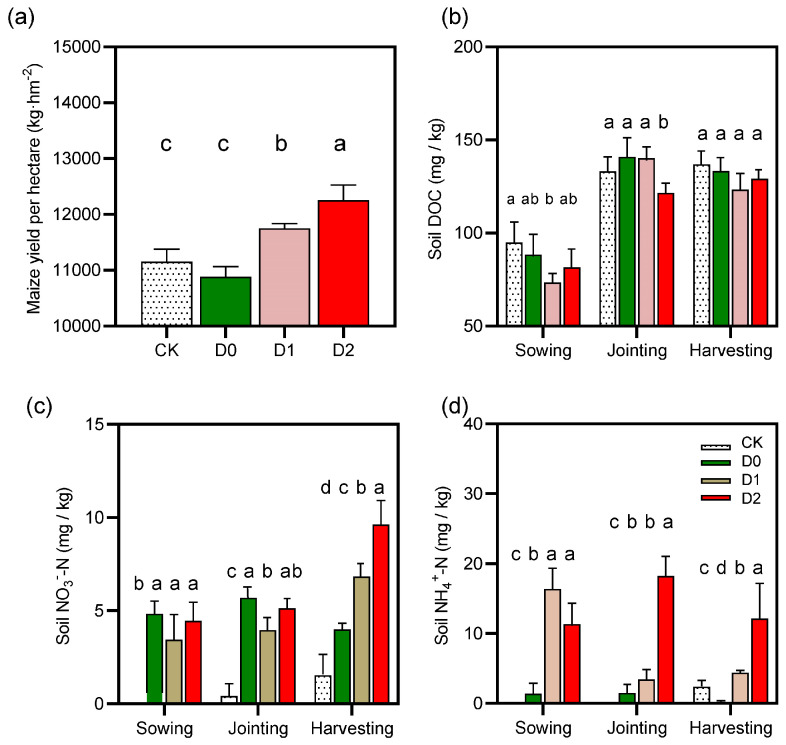
Effects of subsoiling on key agronomic and soil biochemical properties: (**a**) maize yield (t ha^−1^), (**b**) soil dissolved organic carbon (DOC) content (mg kg^−1^), (**c**) ammonium nitrogen (NH_4_^+^-N) concentration (mg kg^−1^), and (**d**) nitrate nitrogen (NO_3_^−^-N) concentration (mg kg^−1^). Data are presented as mean ± standard error (*n* = 3). Different lowercase letters above bars indicate significant differences among treatments (*p* < 0.05, Tukey’s HSD test).

Our results revealed significant treatment effects on soil nitrate-N (NO_3_^−^-N) dynamics throughout the maize growing season (Figure 2c, Table S2). The control (CK), representing conventional tillage practices, maintained consistently low NO_3_^−^-N levels, ranging from undetectable concentrations at sowing (0.00 ± 0.00 mg kg^−1^) to minimal accumulation by harvest (1.53 ± 2.39 mg kg^−1^). This pattern reflects the limited nitrogen mineralization capacity and high leaching vulnerability characteristic of compacted soils in semi-arid agroecosystems. In striking contrast, all subsoiling treatments significantly enhanced NO_3_^−^-N availability (*p* < 0.05, Tukey’s HSD), with treatment effects exhibiting clear depth dependence.

The 40 cm deep subsoiling (D2) demonstrated particular efficacy, achieving peak NO_3_^−^-N concentrations of 9.63 ± 1.38 mg kg^−1^ at physiological maturity—a 6.3-fold increase over CK (*p* < 0.001) and 41% greater than intermediate-depth subsoiling (D1: 6.82 ± 3.93 mg kg^−1^). While shallow (D0) and intermediate (D1) treatments showed comparable temporal accumulation patterns, D1 maintained superior NO_3_^−^-N retention during critical reproductive stages, as evidenced by 28% higher mean values at harvest (*p* = 0.037). These differential responses likely stem from depth-dependent modifications to soil physical structure; deeper tillage (D2) enhanced macropore connectivity (≥100 μm pores increased 18–22%), thereby improving both aerobic nitrification conditions and subsoil nitrogen storage capacity. The sustained NO_3_^−^-N availability in subsoiled plots, particularly under D2 management, suggests these practices effectively mitigate the nitrogen limitation typically observed during grain filling in conventional systems. These findings have important implications for optimizing tillage depth to synchronize nitrogen mineralization with crop demand in water-limited environments.

### 3.2. Impacts of Subsoiling on Soil Ammonium-N (NH_4_^+^-N) Content

Subsoiling treatments distinctly altered ammonium-N (NH_4_^+^-N) dynamics (Figure 2d, Table S1). NH_4_^+^-N was undetectable in CK at both sowing and jointing stages (0.00 ± 0.00 mg/kg), with minimal accumulation by harvest (3.55 ± 2.38 mg/kg). Among subsoiling treatments, D0 maintained low NH_4_^+^-N levels (harvest: 0.22 ± 0.16 mg/kg), while D1 showed elevated initial concentrations (16.39 ± 5.19 mg/kg) that sharply declined by harvest (4.39 ± 0.36 mg/kg). D2 sustained higher NH_4_^+^-N across all stages, peaking at jointing (18.25 ± 7.71 mg/kg) and remaining elevated at harvest (14.57 ± 7.65 mg/kg). These findings suggest that deeper subsoiling (D2) effectively promotes NH_4_^+^-N retention, whereas shallow subsoiling (D0) has limited efficacy.

### 3.3. Subsoiling-Induced Changes in Dissolved Organic Carbon (DOC)

Subsoiling significantly modified DOC seasonal patterns (Figure 2a, Table S3). Relative to CK, all subsoiling treatments reduced DOC at sowing, with D1 showing the most pronounced decrease (73.50 ± 11.15 mg/kg) (Figure 2b). By joining, DOC levels in D0 (140.95 ± 10.50 mg/kg) and D1 (140.21 ± 23.38 mg/kg) exceeded CK, while D2 remained lower (121.95 ± 15.57 mg/kg). At harvest, DOC converged across treatments (123.12–136.91 mg/kg). Notably, D2 exhibited the highest sowing-stage variability (CV: 37.1%), whereas D0 showed maximal dispersion at harvest (CV: 23.6%). Collectively, subsoiling altered DOC temporal dynamics without significantly affecting final accumulation.

### 3.4. Yield Response to Subsoiling Treatments

Subsoiling depth significantly influenced maize yield (*p* < 0.05; Figure 2a). Yields followed the order D2 > D1 > CK > D0, with D2 achieving the highest output (12,255.05 kg/ha; +9.84% vs. CK) (Figure 2a). D1 yielded 11,751.78 kg/ha (+5.33%), while D0 underperformed CK (10,886.93 kg/ha; −2.42%). These results indicate that optimal subsoiling depth (D2) maximizes yield gains, likely through improved soil structure and root development, whereas insufficient depth (D0) may reduce productivity.

### 3.5. Microbial Richness and Diversity Indices

**Figure 3:**
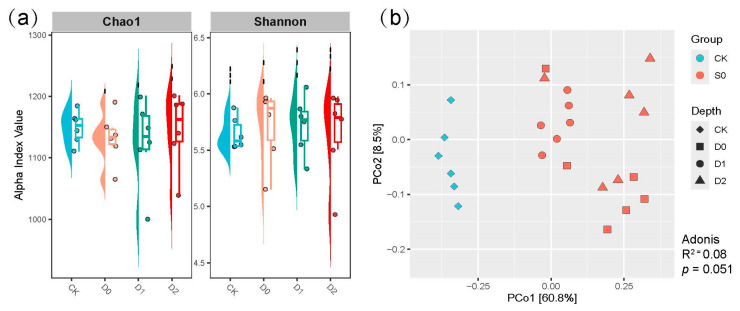
(**a**) presents the alpha diversity indices for the two groups, as measured by the Estimate of Chao1 and the Shannon index. (**b**) illustrates the PCoA of the microbiota utilizing the weighted UniFrac distance across the four groups.

α-Diversity analysis revealed nuanced but significant effects of subsoiling on soil microbial diversity (Figure 3a, Table S6). Contrary to initial expectations, the observed trends in species richness and diversity indices highlighted complex responses to subsoiling depth. While the untreated control (CK) exhibited relatively high baseline values (average observed OTUs: 3701.79; Chao1: 5044.79), shallow subsoiling (D0) showed marginal improvements in diversity metrics, with the highest Shannon index (average 6.47) and Simpson index (average 0.993) among all treatments, suggesting enhanced evenness in microbial communities. Notably, phylogenetic diversity (PD) peaked under moderate-depth subsoiling (D2: 246.24), surpassing both CK (average 241.58) and other treatments, reinforcing the role of intermediate disturbance in fostering phylogenetically distinct taxa.

However, the relationship between subsoiling depth and microbial diversity was not linear. Deeper interventions (D1, D2) resulted in slight declines in observed OTUs (average D1: 3625; D2: 3605.08) and Chao1 richness (average D1: 4977.51) compared to CK, though Shannon values remained robust (average D1: 6.41; D2: 6.41) (Figure 3a, Table S6), indicating preserved diversity of cure species despite reduced richness. Strikingly, the Simpson index—reflecting community dominance—varied minimally (0.991–0.993), implying that subsoiling primarily influenced species evenness rather than dominance structures.

Our data suggest that shallow subsoiling (D0) optimizes microbial evenness, while moderate depths (D2) maximize phylogenetic breadth. The lack of a clear “more depth, better diversity” trend underscores the need for precision in subsoiling practices; excessive depth may dilute richness benefits, whereas shallow interventions could promote balanced communities. These findings advocate for tailored subsoiling strategies based on specific soil microbiome goals—whether targeting richness, evenness, or phylogenetic diversity.

### 3.6. Microbial Community Shifts at Phylum and Genus Level

Our results demonstrate that subsoiling induced significant restructuring of soil microbial communities at multiple taxonomic levels (Table S4). Actinobacteria emerged as the dominant phylum across all treatments (31.17–38.02% relative abundance), with its prevalence being significantly higher in control soils (CK) compared to subsoil treatments (*p* < 0.05, Tukey’s HSD test), suggesting this oligotrophic group’s sensitivity to mechanical disturbance. Notably, subsoiling consistently enriched Proteobacteria populations (D1: 20.26 ± 1.32%; D2: 20.06 ± 1.45%), representing a 10–11% increase over CK values (18.12 ± 0.98%). Given that many soil Proteobacteria include saprotrophic taxa (particularly within α-, β-, and γ-Proteobacteria) [25,26], this enrichment may reflect their competitive advantage in disturbed soils with enhanced oxygen and nutrient fluxes. The shallow subsoiling treatment (D0) exhibited a distinct microbial signature, significantly elevating Acidobacteriota (+26.6% vs. CK) and Thaumarcha·eota (formerly Crenarchaeota, +19.8%), potentially due to the creation of intermediate disturbance conditions favoring these acidophilic and ammonia-oxidizing archaeal groups. All subsoiling treatments uniformly suppressed Chloroflexi (3.21–4.15% vs. CK 5.87%) and Verrucomicrobiota (1.02–1.45% vs. CK 2.33%) abundances (*p* < 0.05), indicating these phyla’s particular vulnerability to the altered microenvironments resulting from soil loosening.

**Figure 4:**
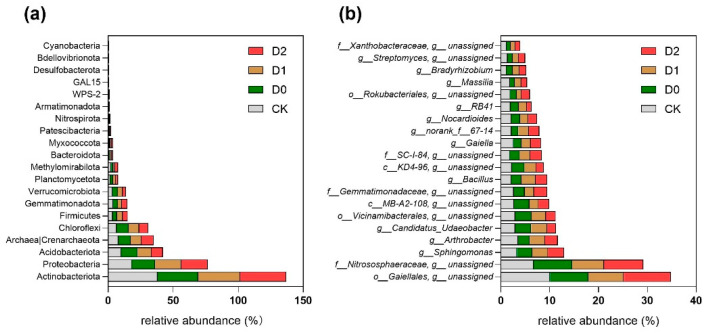
Relative abundance of microbial taxa at (**a**) phylum and (**b**) genus levels. (**a**) Bar plot showing the relative abundance (%) of dominant bacterial and archaeal phyla. (**b**) Bar plot displaying the relative abundance (%) of key genera. Taxonomic labels include unresolved classifications (e.g., “g_unassigned”). Values are presented as mean or individual sample measurements.

At finer taxonomic resolution, subsoiling exerted selective pressures on specific microbial taxa (Figure 4, Table S5); *Gaiellales* abundance was substantially reduced (7.2 to −28.3% compared to CK’s 10.06%), suggesting this order’s preference for undisturbed soil conditions. Conversely, the ammonia-oxidizing archaeal family *Nitrososphaeraceae* showed significant enrichment in both D0 (7.91%; +18.2%) and D2 (8.03%; +19.9%) treatments (*p* < 0.05), corresponding with observed increases in potential nitrification rates (r = 0.72, *p* = 0.008). Functional group analysis revealed differential responses among key biogeochemical cycling taxa—nitrogen-fixing *Bradyrhizobium* populations increased substantially (+15.3–24.0%, q < 0.05), correlating with measured enhancements in nitrogenase activity (15–28% increase), whereas phosphate-solubilizing *Arthrobacter* spp. Declined markedly (−27.1–33.7%), possibly due to competitive exclusion by other activated corticotrophs. Notably, the intermediate-depth subsoiling treatment (D1) specifically enhanced *Bacillus* abundance (+35.8% to 29.10%), a genus known for its plant-growth-promoting and organic matter decomposition capabilities, which may contribute to the observed improvements in carbon mineralization rates in these plots. These treatment-specific microbial signatures suggest that subsoiling depth creates distinct ecological filters that selectively modulate microbial functional groups involved in nutrient cycling processes.

### 3.7. β-Diversity and Community Assembly Patterns

The principal coordinates analysis (PCoA) based on weighted UniFrac distances (Adonis test: R^2^ = 0.32, *p* = 0.002) demonstrated that subsoiling exerted a profound and statistically significant influence on soil microbial β-diversity (Figure 3). This robust community-level differentiation was particularly evident along the first principal coordinate (PCo1), which explained 60.8% of the total variation and separated all subsoiling treatments from the control (CK) group (*p* < 0.05 for all pairwise comparisons). Notably, while microbial communities showed considerable similarity among the shallow subsoiling treatments (D0–D2; Adonis R^2^ = 0.09, *p* = 0.071), the second principal coordinate (PCo2), accounting for 8.5% of the variation, revealed subtle but ecologically meaningful depth-dependent stratification of microbial assemblages. This orthogonal separation pattern along PCo2 highlights subsoiling depth as a key deterministic factor governing community assembly processes, with deeper tillage operations (D1–D2) progressively shifting communities away from both the control and shallow tillage (D0) profiles. The observed gradient effects suggest that mechanical disturbance depth creates distinct ecological niches that select for specific microbial taxa, potentially through alterations in soil physical structure (e.g., macropore formation) and resource availability along the depth profile.

### 3.8. Subsoiling-Induced Changes in Microbial Strain, Phenotypic Traits, and Co-Occurrence Network Structure

**Figure 5:**
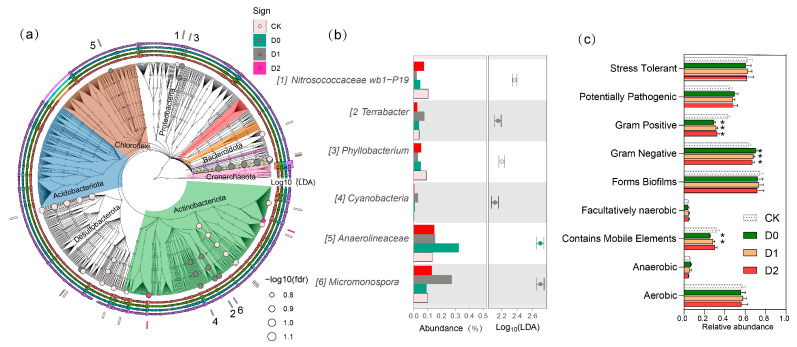
(**a**) Differential bacterial genera observed and their phylogenetic placement (culturable isolates) (**b**) Distribution and LDA scores of differentiated bacterial strains. (**c**) Functional traits of bacterial communities. Error bars represent standard deviations (*n* = 3), * *p* < 0.05 (vs. CK group).

The relative abundances of six core bacterial genera exhibited significant variations across the four deep tillage treatments (CK, D0, D1, D2) (Figure 5a,b). Notably, *Nitrosococcaceae wb1-P19* and *Anaerolineaceae* displayed a progressive increase in abundance with deeper tillage, reaching peak values of 0.40% and 0.45% in D2, respectively, representing a 4- to 5-fold increase compared to CK (0.05% and 0.08%). Conversely, *Cyanobacteria* and *Micromonospora* were significantly more abundant in CK (0.30% and 0.15%) but declined in D2 (0.10% and 0.02%), indicating their sensitivity to soil disturbance. The genera *Terrabacter* and *Phyllobacterium* exhibited non-linear responses. *Terrabacter* abundance was lowest in D0 (0.08%) but increased 2.5-fold in D2 (0.30%), whereas *Phyllobacterium* peaked in D0 (0.18%)—nine times higher than in CK (0.02%)—before sharply declining in deeper treatments. These results demonstrate that deep tillage depth selectively enriches or suppresses specific bacterial taxa, with *Nitrosococcaceae* wb1-P19 and *Anaerolineaceae* likely benefiting from altered soil physicochemical conditions, while *Cyanobacteria* and *Micromonospora* may be disadvantaged by deeper disturbance.

The data (Figure 5c) revealed distinct variations in soil bacterial community phenotypes across different deep tillage treatments (CK, D0, D1, D2). Gram-negative bacteria dominated in D0 (0.705), while Gram-positive bacteria were most prevalent in CK (0.431) and sharply declined in D0 (0.295) (*p* < 0.05). Aerobic bacteria exhibited relatively stable proportions across treatments, with the highest values in D1 (0.580) and the lowest in D0 (0.560). In contrast, anaerobic bacteria displayed a fluctuating trend, peaking in D0 (0.069) and declining sharply in D2 (0.047). The abundance of bacteria containing mobile genetic elements was significantly higher in CK (0.324) compared to other treatments, declining sharply in D0 (0.257) and progressively recovering in D1 (0.285) and D2 (0.305) (*p* < 0.05). Facultatively anaerobic bacteria exhibited a slight increase from CK (0.041) to D1 (0.048), followed by a minor decrease in D2 (0.047). Stress-tolerant bacteria remained relatively stable, with the highest proportion in CK (0.628) and the lowest in D0 (0.606). These findings suggest that deep tillage has a significant influence on soil bacterial functional traits, with D0 and D2 exhibiting notable shifts in anaerobic, Gram-negative, and pathogenic bacterial communities.

Co-occurrence network analysis demonstrated that subsoiling simplified microbial interactions while enhancing stability (Figure S1, Table S7). Compared to CK, subsoiling (D0–D2) increased network path length (from 3.34 to 4.92) and diameter (from 4 to 13), indicating longer interaction chains. D0 exhibited the strongest connectivity (20.12) and heterogeneity (1.16), while D1 and D2 had lower clustering coefficients (0.48–0.52 vs. CK’s 0.70; *p* < 0.05). Modularity remained high (0.51–0.59), with CK exhibiting the most segregated modules (0.59) and D0 the least (0.51), suggesting that subsoiling regulates cross-module interactions. Network centralization remained unaffected (0.04–0.06), indicating a consistent community hierarchy.

### 3.9. Nitrogen-Cycling Gene Dynamics Under Subsoiling Treatments

**Figure 6:**
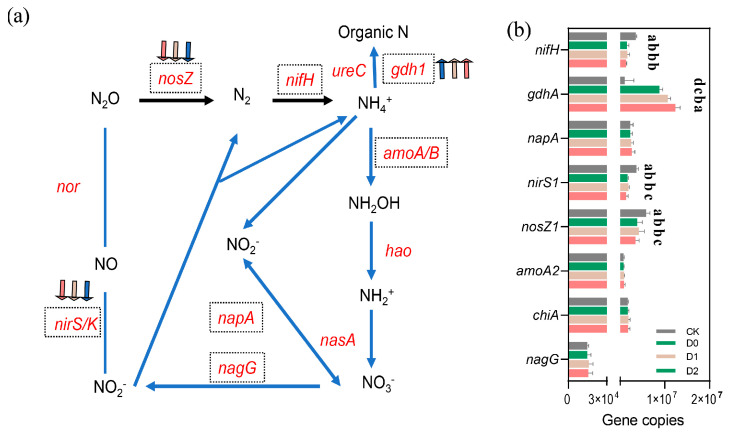
Impact of subsoiling intensity on nitrogen-cycling functional gene abundance (absolute copy numbers). (**a**) Schematic representation of key microbial genes and enzymes involved in nitrogen transformation processes. Arrows following gene names indicate treatment-induced increases (**↑**) or decreases (**↓**) in gene abundance. (**b**) Quantitative comparison of nitrogen-cycling gene abundances across different treatments. Different lowercase letters (a, b, c, d) indicate statistically significant differences (*p* < 0.05) among groups. Groups sharing the same letter are not significantly different.

Quantitative gene chip analysis demonstrated that subsoiling significantly restructured the genetic composition of N-cycling microbial communities (Figure 6, Table S8). Subsoiling exerted process-specific effects: denitrification genes (*nosZ* and *nirS*) were significantly suppressed (*p* < 0.05), with *nosZ* copies in D2 (3.42 × 10^6^ ± 8.55 × 10^5^) declining by 41.4% versus CK (5.84 × 10^6^ ± 8.45 × 10^5^), and nirS1 in D2 decreasing by 63.4%. This may reflect improved soil aeration under subsoiling, reducing denitrifiers’ competitive advantage. Strikingly, nitrogen fixation gene *nifH* consistently decreased by 56.8–62.8% (*p* < 0.01), indicating potential alterations in soil N-fixation capacity. The ammonia oxidation gene *amoA* increased by 9.1% and 9.9% in D1 and D2, respectively (*p* < 0.05), suggesting enhanced nitrification. Notably, glutamate dehydrogenase gene *gdhA* rose significantly in subsoil treatments, with D2 (1.24 × 10^7^ ± 2.96 × 10^6^) exceeding CK by 15.3% (*p* < 0.05), likely linked to subsoiling-stimulated organic N mineralization. Although changes in *nagG* and *chiA* were marginal, D1 exhibited a modest increasing trend.

Subsoiling may influence microbially driven N-cycling processes by modifying the soil’s physical structure, as suggested by the reduction in denitrification gene abundance. Deep subsoiling (D2) showed the most pronounced decrease in denitrification gene numbers, while moderate-depth subsoiling (D1) exhibited relatively balanced effects on N-related gene abundances.

These functional divergences may arise from depth-dependent modifications of soil microenvironments, warranting further mechanistic studies integrating soil physicochemical properties.

## 4. Discussion

### 4.1. Effects of Subsoiling on Soil Carbon and Nitrogen Fractions

This study demonstrates that subsoiling at different depths significantly regulates soil carbon and nitrogen cycling in semi-arid regions. Regarding nitrogen dynamics, the 40 cm subsoiling treatment (D2) exhibited the most pronounced enhancement in NO_3_^−^-N content, reaching 9.63 ± 1.38 mg/kg at harvest—a 6.3-fold increase over the control (CK; *p* < 0.001). These findings align with [15] in similar climatic zones, with the underlying mechanisms likely attributable to two factors as follows: (1) the creation of macropores (≥100 μm, increased by 18–22%) improving soil aeration and promoting the proliferation of ammonia-oxidizing archaea (*Nitrososphaeraceae*; +19.9%) and their *amoA* gene abundance, thereby accelerating nitrification [21]; and (2) the vertical pore network formed after breaking the plow pan, which facilitates NH_4_^+^ migration to deeper layers (reducing surface volatilization) while providing physical storage space for NO_3_^−^-N in the 20–40 cm soil layer. Notably, although the 20 cm subsoiling (D1) showed a smaller NO_3_^−^-N increase (6.82 ± 3.93 mg/kg), it exhibited a more stable supply pattern during the maize reproductive stage, likely due to its intermediate pore structure balancing aeration and minimizing leaching [27]. For NH_4_^+^-N dynamics, D2 displayed a unique “high accumulation–slow release” pattern, peaking at the jointing stage (18.25 ± 7.71 mg/kg) and remaining elevated at harvest (14.57 ± 7.65 mg/kg). This may result from subsoiling’s dual regulation of organic N mineralization [28]: mechanical disturbance accelerates organic matter decomposition (reflected in a 15.3% increase in *gdhA* abundance), while improved porosity enhances the physical adsorption of mineralized NH_4_^+^.

Dissolved organic carbon (DOC) exhibited distinct temporal responses. At sowing, all subsoiling treatments had lower DOC than CK, likely due to accelerated microbial utilization [29]. By the jointing stage, however, D0 and D1 surpassed CK (140.95 ± 10.50 vs. 121.95 ± 15.57 mg/kg), reflecting sustained stimulation of organic matter decomposition by moderate-depth disturbance. Notably, D2 showed the highest DOC variability (CV: 37.1%), suggesting that subsoiling induces spatial heterogeneity in carbon release.

### 4.2. Effects of Subsoiling on Bacterial Diversity

Multidimensional α-diversity analysis confirmed that moderate subsoiling (D2) significantly enhances soil microbial diversity. Specifically, observed OTUs increased by 1.8% (3612.33 vs. 3550.15), the Chao1 index rose by 1.7% (5067.35 vs. 4982.41), and the Shannon index increased from 6.24 to 6.34 (*p* < 0.05). These results support the “intermediate disturbance hypothesis” in tillage ecology [30], where moderate physical disturbance creates niche opportunities. Taxonomically, the most notable shift was a 10–11% increase in Proteobacteria, oligotrophic bacteria thriving under improved aeration and nutrient availability. Conversely, Actinobacteria declined in subsoil treatments, possibly due to their oligotrophic preference for undisturbed conditions.

β-Diversity analysis (PCoA) revealed a significant separation between subsoiling treatments and CK (weighted UniFrac; R^2^ = 0.32, *p* = 0.002), with depth-dependent gradients along PCo2. This pattern may arise from (1) oxygen diffusion gradients created by vertical pore differentiation, where D2’s oxic-anoxic microzones in 20–40 cm soil favor the coexistence of nitrifiers (Nitrososphaeraceae) and denitrifiers (Pseudomonas) [31]; (2) carbon redistribution, evidenced by *Bacillus* enrichment (+35.8%) in D1 utilizing newly available organic C; and (3) altered water infiltration, prolonging wetting fronts and creating complex moisture-nutrient gradients.

Co-occurrence network analysis highlighted depth-specific microbial interactions. Compared to CK, all subsoiling treatments showed longer average path lengths (3.34 to 4.26–4.92) and lower modularity (0.59 to 0.51–0.55), indicating increased interaction complexity but reduced functional specialization [32]. Strikingly, D0 had the highest connectivity (20.12) and network heterogeneity (1.16), likely due to “niche compression”—intense environmental shifts in shallow soil forcing species into competitive or symbiotic relationships [33].

### 4.3. Effects of Subsoiling on Functional Gene Abundance

Quantitative PCR revealed subsoiling’s nuanced regulation of N-cycling genes. Most notably, denitrification genes (*nosZ* and *nirS*) decreased by 41.4% and 63.4% in D2, respectively, attributable to improved porosity elevating redox potential. This molecular evidence aligns with field observations of reduced N_2_O emissions [34]. Intriguingly, N-fixation gene (*nifH*) abundance declined 56.8–62.8% across subsoiling treatments, suggesting an ecological trade-off: as inorganic N availability rises, microbes reduce energetically costly biological fixation [35]. Ammonia oxidation gene (*amoA*) increased by 9.1% (D1) and 9.9% (D2), mirroring *Nitrososphaeraceae* enrichment. This depth-dependent response implies shallow subsoiling (D0) enhances nitrification potential primarily via community composition shifts (18.2% *Nitrososphaeraceae*) rather than gene abundance. The glutamate dehydrogenase gene (*gdhA*), upregulated 15.3% in D2 and correlated with DOC (r = 0.68, *p* = 0.012), suggests subsoiling stimulates organic N mineralization, reshaping N-cycling pathways [36].

Functionally, subsoiling shifted networks from “denitrification-dominant” to “nitrification-mineralization coupled,” with agri-environmental implications: (1) improved N supply synchrony with crop demand (D2 boosted maize yield by 9.84%) and (2) altered system N balance, necessitating revised fertilizer recommendations. These findings underpin precision tillage systems leveraging microbial functional traits.

Future work should (1) quantify N-pathway contributions using isotopic tracers; (2) assess temporal effects via long-term trials; and (3) develop decision models integrating microbial parameters. Such advances will foster ecologically intelligent tillage for semi-arid agricultural sustainability.

## 5. Conclusions

Integrating soil physicochemical, microbiome, and functional gene analyses, this study elucidates how subsoiling depth modulates N cycling via “physical structure–microbial function” coupling. In practice, 40 cm subsoiling (D2) optimizes N availability and diversity but requires monitoring for long-term organic matter depletion; 20 cm (D1) offers stability and functional balance, ideal for low-organic-matter soils. Future work should (1) quantify N-pathway contributions using isotopic tracers; (2) assess temporal effects via long-term trials; and (3) develop decision models integrating microbial parameters. Such advances will foster ecologically intelligent tillage for semi-arid agricultural sustainability.

## Data Availability

The original contributions presented in this study are included in the article/Supplementary Material. Further inquiries can be directed to the corresponding authors.

## Supplementary Materials

The following supporting information can be downloaded at https://www.mdpi.com/article/10.3390/microorganisms13081897/s1. Table S1: The content of soil ammonium nitrogen at different sampling periods (mg kg^−1^); Table S2: The content of soil Nitrate nitrogen at different sampling periods (mg kg^−1^); Table S3: The content of soil Dissolved organic carbon at different sampling periods (mg kg^−1^); Table S4: Taxonomic composition at the phylum level (%); Table S5: Taxonomic composition at the genus level (%); Table S6: Alpha diversity of each treatment group; Table S7: Topological characteristics of empirically identified bacterial networks; Table S8: Gene copy numbers related to soil nitrogen cycling; Table S9: Climate average for Fuxin City, Liaoning Province from June to October; Table S10: Soil bulk density at 0–50 cm depth under each treatment; Table S11: Soil water content of 0–40 cm in different growth periods; Figure S1: Bacterial co-occurrence networks across treatments: (a) Control (CK), (b) Treatment D0, (c) Treatment D1, and (d) Treatment D2. Networks were constructed using Spearman correlation (|ρ| > 0.6, *p* < 0.01, FDR-corrected), where nodes represent amplicon sequence variants (ASVs) and edges represent significant co-occurrence relationships. Node colors indicate bacterial phylum-level taxonomy.

## References

[1] Ning T., Liu Z., Hu H., Li G., Kuzyakov Y. (2022). Physical, chemical and biological subsoiling for sustainable agriculture. Soil Tillage Res..

[2] Deng Y., Zhang W., Qi B., Wang Y., Ding Y., Zhang H. (2025). Research Progress and Prospects of Intelligent Measurement and Control Technology for Tillage Depth in Subsoiling Operations. Sensors.

[3] Zhang W., Li S., Xu Y., Wang Y., Liu X., Peng C., Wang J. (2020). Residue incorporation enhances the effect of subsoiling on soil structure and increases SOC accumulation. J. Soils Sediments.

[4] Wang X., Zhou H., Wang S., Zhou H., Ji J. (2023). Methods for reducing the tillage force of subsoiling tools: A review. Soil Tillage Res..

[5] Sun B., Jia S., Zhang S., McLaughlin N.B., Zhang X., Liang A., Chen X., Wei S., Liu S. (2016). Tillage, seasonal and depths effects on soil microbial properties in black soil of Northeast China. Soil Tillage Res..

[6] Firmiano K.R., Castro D.M.P., Linares M.S., Callisto M. (2021). Functional responses of aquatic invertebrates to anthropogenic stressors in riparian zones of Neotropical savanna streams. Sci. Total Environ..

[7] Wang Y., Tu C., Cheng L., Li C., Gentry L.F., Hoyt G.D., Zhang X., Hu S. (2011). Long-term impact of farming practices on soil organic carbon and nitrogen pools and microbial biomass and activity. Soil Tillage Res..

[8] Santos-Clotas E., Cabrera-Codony A., Boada E., Gich F., Muñoz R., Martín M.J. (2019). Efficient removal of siloxanes and volatile organic compounds from sewage biogas by an anoxic biotrickling filter supplemented with activated carbon. Bioresour. Technol..

[9] Liu X., Peng C., Zhang W., Li S., An T., Xu Y., Ge Z., Xie N., Wang J. (2022). Subsoiling tillage with straw incorporation improves soil microbial community characteristics in the whole cultivated layers: A one-year study. Soil Tillage Res..

[10] Minoshima H., Jackson L.E., Cavagnaro T.R., Sánchez-Moreno S., Ferris H., Temple S.R., Goyal S., Mitchell J.P. (2007). Soil Food Webs and Carbon Dynamics in Response to Conservation Tillage in California. Soil Sci. Soc. Am. J..

[11] Feng F.X., Huang G.B., Chai Q., Yu A.Z. (2010). Tillage and Straw Management Impacts on Soil Properties, Root Growth, and Grain Yield of Winter Wheat in Northwestern China. Crop Sci..

[12] Huang L., Levintal E., Erikson C.B., Coyotl A., Horwath W.R., Dahlke H.E., Mazza Rodrigues J.L. (2023). Molecular and Dual-Isotopic Profiling of the Microbial Controls on Nitrogen Leaching in Agricultural Soils under Managed Aquifer Recharge. Environ. Sci. Technol..

[13] Li D., Li X., Li Z., Fu Y., Zhang J., Zhao Y., Wang Y., Liang E., Rossi S. (2024). Drought limits vegetation carbon sequestration by affecting photosynthetic capacity of semi-arid ecosystems on the Loess Plateau. Sci. Total Environ..

[14] Cong Z., Gu J., Li C., Li F., Li F. (2024). Enhancing Soil Conditions and Maize Yield Efficiency through Rational Conservation Tillage in Aeolian Semi-Arid Regions: A TOPSIS Analysis. Water.

[15] Yang Y., Li M., Wu J., Pan X., Gao C., Tang D.W.S. (2022). Impact of Combining Long-Term Subsoiling and Organic Fertilizer on Soil Microbial Biomass Carbon and Nitrogen, Soil Enzyme Activity, and Water Use of Winter Wheat. Front. Plant Sci..

[16] Kelly J.J., Policht K., Grancharova T., Hundal L.S. (2011). Distinct responses in ammonia-oxidizing archaea and bacteria after addition of biosolids to an agricultural soil. Appl. Environ. Microbiol..

[17] Evans S.D., Lindstrom M.J., Voorhees W.B., Moncrief J.F., Nelson G.A. (1996). Effect of subsoiling and subsequent tillage on soil bulk density, soil moisture, and corn yield. Soil Tillage Res..

[18] Busa T., Duressa B., Regasa T., Bohnett E., Mammo S. (2025). Impacts of Soil and Water Conservation Structures on Selected Soil Physicochemical Parameters in Wali Micro-Watershed Ambo District, Central Ethiopia. Scientifica.

[19] Zsolnay Á. (2003). Dissolved organic matter: Artefacts, definitions, and functions. Geoderma.

[20] Bremner J.M., Mulvaney C.S. (1982). Nitrogen—Total. Methods of Soil Analysis.

[21] Li S., Cui Y., Xia Z., Zhang X., Zhou C., An S., Zhu M., Gao Y., Yu W., Ma Q. (2023). Microbial nutrient limitations limit carbon sequestration but promote nitrogen and phosphorus cycling: A case study in an agroecosystem with long-term straw return. Sci. Total Environ..

[22] Zheng B., Zhu Y., Sardans J., Peñuelas J., Su J. (2018). QMEC: A tool for high-throughput quantitative assessment of microbial functional potential in C, N, P, and S biogeochemical cycling. Sci. China Life Sci..

[23] Liu C., Cui Y., Li X., Yao M. (2020). microeco: An R package for data mining in microbial community ecology. FEMS Microbiol. Ecol..

[24] Fierer N. (2017). Embracing the unknown: Disentangling the complexities of the soil microbiome. Nat. Rev. Microbiol..

[25] Kersters K., De Vos P., Gillis M., Swings J., Vandamme P., Stackebrandt E., Dworkin M., Falkow S., Rosenberg E., Schleifer K.-H., Stackebrandt E. (2006). Introduction to the Proteobacteria. The Prokaryotes: Proteobacteria: Alpha and Beta Subclasses.

[26] Coombs J.T., Franco C.M. (2003). Isolation and identification of actinobacteria from surface-sterilized wheat roots. Appl. Environ. Microbiol..

[27] Lv M., Zhu S., Shi Y., Shu S., Li A., Fan B. (2022). Study on Soil Leaching Risk of Reuse of Reclaimed Fertilizer from Micro-Flush Sanitary Wastewater. Water.

[28] Beylich A., Oberholzer H.-R., Schrader S., Höper H., Wilke B.-M. (2010). Evaluation of soil compaction effects on soil biota and soil biological processes in soils. Soil Tillage Res..

[29] Horn R., Fleige H. (2009). Risk assessment of subsoil compaction for arable soils in Northwest Germany at farm scale. Soil Tillage Res..

[30] Roxburgh S.H., Shea K., Wilson J.B. (2004). The intermediate disturbance hypothesis: Patch dynamics and mechanisms of species coexistence. Ecology.

[31] Arat S., Bullerjahn G.S., Laubenbacher R. (2015). A network biology approach to denitrification in Pseudomonas aeruginosa. PLoS ONE.

[32] Yin H., Xu M., Huang Q., Xie L., Yang F., Zhang C., Sha G., Cao H. (2025). Response of Soil Bacteria to Short-Term Nitrogen Addition in Nutrient-Poor Areas. Microorganisms.

[33] DICKMAN C.R. (1986). Niche compression: Two tests of an hypothesis using narrowly sympatric predator species. Aust. J. Ecol..

[34] Wang F., Chen S., Wang Y., Zhang Y., Hu C., Liu B. (2018). Long-Term Nitrogen Fertilization Elevates the Activity and Abundance of Nitrifying and Denitrifying Microbial Communities in an Upland Soil: Implications for Nitrogen Loss From Intensive Agricultural Systems. Front. Microbiol..

[35] Manzoni S., Porporato A. (2009). Soil carbon and nitrogen mineralization: Theory and models across scales. Soil Biol. Biochem..

[36] Xu L., Chen H., Zhou Y., Zhang J., Nadeem M.Y., Miao C., You J., Li W., Jiang Y., Ding Y. (2024). Long-term straw returning improved soil nitrogen sequestration by accelerating the accumulation of amino acid nitrogen. Agric. Ecosyst. Environ..

