# Supplementary material for: Subsoiling-Induced Shifts in Nitrogen Dynamics and Microbial Community Structure in Semi-Arid Rainfed Maize Agroecosystems
Source: Microorganisms. 2025 Aug 14;13(8):1897. doi: 10.3390/microorganisms13081897 (PMC12388414; doi:10.3390/microorganisms13081897)
Supplement: Supplementary file 1 [file microorganisms-13-01897-s001.zip › microorganisms-3717401-supplementary.pdf]

Table S1. The content of soil ammonium nitrogen at different sampling periods (mg kg^{-1}).

	CK						Average
Sowing	0	0	0	0	0	0	1. 0.00 ± 0.00
Jointing	0	0	0	0	0	0	2. 0.00 ± 0.00
Harvesting	0	0	4.66	4.74	0.07	4.74	3. 3.55 ± 2.38
						D0	
Sowing	0.56	2.95	0.3	0.62	3.6	0.36	4. 1.40 ± 1.49
Jointing	0.05	0.19	4.33	0	0.06	4.19	5. 1.47 ± 2.09
Harvesting	0.42	0.18	0.08	0.41	0.13	0.08	6. 0.22 ± 0.16
						D1	
Sowing	4.64	33.97	6.06	4.5	42.29	6.88	7. 16.39 ± 16.69
Jointing	2.52	8.61	0	2	7.27	0	8. 3.40 ± 3.53
Harvesting	4.53	4.21	4.62	4.27	3.88	4.84	9. 4.·39 ± 0.36
						D2	
Sowing	0	17.22	16.57	0	17.59	16.68	10. 14.41 ± 7.93
Jointing	25.73	23.72	8.77	22.23	20.35	8.68	11. 18.25 ± 7.71
Harvesting	0	20.06	19.08	0	17.4	16.3	12. 14.57 ± 7.65

Table S2. The content of soil Nitrate nitrogen at different sampling periods (mg kg^{-1}).

	CK						Average
Sowing	0	0	0	0	0	0	0.00±0.00
Jointing	0	1.1216	1.4101	0	0	0	0.42±0.69
Harvesting	0	0	4.6	0	0	4.6	1.53±2.39
						D0	
Sowing	4.503	3.767	4.797	4.335	3.057	5.098	4.26±0.74
Jointing	2.475	7.626	9.081	2.643	6.997	5.321	5.69±2.51
Harvesting	4.464	3.965	3.674	4.364	3.713	3.793	3.99±0.45
						D1	
Sowing	5.432	1.481	3.169	4.442	2.923	3.179	3.44±1.48
Jointing	3.042	1.093	4.222313	6.06	5.05	4.294764	3.96±1.76
Harvesting	5.328	6.1525	12.0105	0.331	8.1225	9.0075	6.82±3.93
						D2	
Sowing	4.80	5.97	6.04	0.00	5.05	4.87	4.46±2.21
Jointing	8.85	3.94	2.66	6.17	5.50	3.66	5.13±2.24
Harvesting	8.96	9.72	11.66	8.00	10.39	9.07	9.63±1.38

Table S3. The content of soil Dissolved organic carbon at different sampling periods (mg kg^{-1}).

	CK						Average
Sowing	104.729	61.809	91.804	115.073	83.035	113.192	94.77 ± 20.44
Jointing	139.017	129.846	122.608	109.846	142.103	155.630	133.18 ± 15.62
Harvesting	134.542	111.024	139.378	114.812	163.080	158.613	136.91 ± 20.61
	D0						
Sowing	76.658	66.392	83.367	71.090	108.635	112.652	86.63 ± 19.41
Jointing	142.322	133.604	146.314	127.918	157.141	138.421	140.95 ± 10.50
Harvesting	118.465	120.662	188.406	103.561	110.338	158.735	133.20 ± 31.36
	D1						
Sowing	64.414	77.992	81.413	57.798	71.983	87.397	73.50 ± 11.15
Jointing	136.718	167.178	145.086	134.983	154.267	103.020	140.21 ± 23.38
Harvesting	99.514	154.068	106.771	111.702	162.442	105.226	123.12 ± 25.65
	D2						
Sowing	63.630	53.521	67.375	60.947	127.713	116.175	81.56 ± 30.25
Jointing	130.216	119.523	91.787	130.447	123.208	134.495	121.95 ± 15.57
Harvesting	113.437	130.977	126.116	114.152	163.519	127.176	129.23 ± 17.83

Table S4. Taxonomic composition at the phylum level (numbers represent a relative abundances of the top phyla %).

taxa	CK						D0					
Actinobacteriota	0.291	0.480	0.419	0.357	0.346	0.388	0.329	0.316	0.297	0.319	0.295	0.315
Proteobacteria	0.103	0.119	0.121	0.254	0.236	0.259	0.171	0.070	0.112	0.235	0.242	0.245
Acidobacteriota	0.182	0.066	0.115	0.071	0.086	0.081	0.116	0.168	0.140	0.124	0.098	0.115
Archaea|Crenarchaeota	0.142	0.074	0.113	0.077	0.045	0.022	0.091	0.182	0.169	0.028	0.071	0.026
Chloroflexi	0.080	0.050	0.058	0.063	0.081	0.063	0.097	0.072	0.085	0.099	0.093	0.100
Firmicutes	0.023	0.024	0.027	0.048	0.053	0.040	0.030	0.012	0.037	0.031	0.045	0.035
Gemmatimonadota	0.029	0.040	0.039	0.041	0.044	0.037	0.031	0.027	0.037	0.030	0.046	0.040
Verrucomicrobiota	0.067	0.028	0.033	0.021	0.027	0.028	0.047	0.052	0.050	0.036	0.023	0.028
Planctomycetota	0.009	0.004	0.005	0.015	0.027	0.036	0.016	0.007	0.009	0.039	0.024	0.036
Methylomirabilota	0.035	0.068	0.029	0.006	0.003	0.002	0.022	0.047	0.027	0.001	0.002	0.002
Bacteroidota	0.003	0.002	0.003	0.016	0.012	0.015	0.005	0.001	0.003	0.008	0.017	0.020
Myxococcota	0.009	0.015	0.009	0.008	0.008	0.007	0.009	0.009	0.007	0.006	0.007	0.007
Patescibacteria	0.001	0.001	0.001	0.004	0.008	0.007	0.007	0.000	0.001	0.012	0.007	0.006
Nitrospirota	0.006	0.011	0.009	0.003	0.003	0.002	0.004	0.008	0.006	0.002	0.002	0.002
Armatimonadota	0.002	0.001	0.003	0.004	0.004	0.003	0.003	0.001	0.002	0.007	0.004	0.005
WPS-2	0.000	0.000	0.000	0.000	0.003	0.002	0.005	0.000	0.000	0.008	0.003	0.002
GAL15	0.001	0.002	0.003	0.000	0.000	0.000	0.002	0.010	0.001	0.000	0.000	0.000
Desulfobacterota	0.003	0.005	0.004	0.001	0.000	0.000	0.001	0.004	0.002	0.000	0.000	0.000
Bdellovibrionota	0.001	0.000	0.000	0.002	0.003	0.002	0.001	0.000	0.000	0.001	0.003	0.004
Cyanobacteria	0.000	0.000	0.000	0.000	0.001	0.001	0.002	0.000	0.000	0.002	0.009	0.003
	D1						D2					
Actinobacteriota	0.296	0.346	0.398	0.317	0.260	0.323	0.385	0.348	0.375	0.358	0.343	0.312
Proteobacteria	0.134	0.180	0.131	0.312	0.203	0.256	0.232	0.127	0.085	0.245	0.257	0.257
Acidobacteriota	0.142	0.137	0.049	0.057	0.165	0.091	0.077	0.085	0.089	0.081	0.094	0.095
Crenarchaeota	0.121	0.085	0.174	0.022	0.036	0.060	0.049	0.195	0.215	0.025	0.020	0.054
Chloroflexi	0.086	0.077	0.091	0.067	0.088	0.086	0.059	0.056	0.049	0.097	0.075	0.083
Firmicutes	0.043	0.037	0.028	0.078	0.051	0.036	0.035	0.036	0.016	0.035	0.063	0.040
Gemmatimonadota	0.035	0.029	0.020	0.036	0.038	0.039	0.048	0.048	0.025	0.034	0.051	0.041
Verrucomicrobiota	0.058	0.033	0.042	0.016	0.059	0.027	0.021	0.024	0.027	0.025	0.024	0.020
Planctomycetota	0.016	0.012	0.006	0.019	0.056	0.028	0.009	0.006	0.003	0.029	0.024	0.021
Methylomirabilota	0.023	0.022	0.022	0.000	0.005	0.001	0.030	0.031	0.065	0.002	0.000	0.007

Bacteroidota	0.004	0.007	0.007	0.023	0.009	0.016	0.006	0.004	0.001	0.011	0.011	0.021
Myxococcota	0.008	0.011	0.008	0.015	0.008	0.007	0.011	0.010	0.014	0.010	0.008	0.008
Patescibacteria	0.005	0.000	0.003	0.012	0.004	0.010	0.013	0.002	0.000	0.012	0.009	0.006
Nitrospirota	0.004	0.005	0.003	0.002	0.002	0.002	0.005	0.008	0.009	0.001	0.002	0.002
Armatimonadota	0.002	0.001	0.001	0.003	0.004	0.005	0.003	0.001	0.001	0.007	0.004	0.006
WPS-2	0.005	0.000	0.001	0.009	0.001	0.004	0.002	0.000	0.000	0.012	0.003	0.001
GAL15	0.001	0.000	0.004	0.000	0.000	0.000	0.001	0.005	0.013	0.000	0.000	0.000
Desulfobacterota	0.002	0.002	0.003	0.000	0.001	0.000	0.002	0.002	0.006	0.000	0.000	0.001
Bdellovibrionota	0.001	0.001	0.001	0.003	0.002	0.003	0.001	0.001	0.000	0.002	0.002	0.004
Cyanobacteria	0.001	0.001	0.001	0.002	0.001	0.001	0.000	0.000	0.000	0.003	0.001	0.006

Table S5. Taxonomic composition at the genus level (%) (numbers represent a relative abundances of the top genera %).

TAXA	CK						D0					
o__Gaiellales, g__ unassigned	0.076	0.177	0.140	0.074	0.078	0.059	0.099	0.089	0.090	0.074	0.075	0.046
f__Nitrososphaeraceae, g__ unassigned	0.129	0.072	0.107	0.056	0.028	0.010	0.075	0.177	0.156	0.013	0.042	0.012
g__Sphingomonas	0.014	0.010	0.014	0.058	0.056	0.046	0.021	0.007	0.011	0.040	0.053	0.055
g__Arthrobacter	0.013	0.005	0.013	0.033	0.047	0.102	0.018	0.005	0.008	0.046	0.027	0.038
g__Candidatus_Udaeobacter	0.062	0.026	0.030	0.017	0.021	0.020	0.041	0.048	0.045	0.028	0.016	0.019
o__Vicinamibacterales, g__ unassigned	0.051	0.016	0.030	0.019	0.028	0.031	0.023	0.051	0.040	0.026	0.022	0.039
c__MB-A2-108, g__ unassigned	0.051	0.028	0.067	0.005	0.007	0.002	0.029	0.089	0.047	0.010	0.006	0.009
f__Gemmatimonadaceae, g__ unassigned	0.023	0.036	0.035	0.024	0.022	0.019	0.020	0.025	0.031	0.015	0.023	0.018
g__Bacillus	0.014	0.012	0.013	0.030	0.035	0.025	0.020	0.006	0.026	0.020	0.030	0.022
c__KD4-96, g__ unassigned	0.040	0.020	0.027	0.016	0.018	0.014	0.025	0.031	0.039	0.016	0.015	0.029
f__SC-I-84, g__ unassigned	0.008	0.012	0.010	0.028	0.025	0.026	0.023	0.005	0.012	0.031	0.025	0.026
g__Gaiella	0.028	0.058	0.048	0.012	0.007	0.003	0.021	0.033	0.029	0.004	0.006	0.004
g__norank_f__67-14	0.020	0.047	0.031	0.015	0.010	0.008	0.017	0.021	0.017	0.008	0.013	0.009
g__Nocardioides	0.005	0.009	0.003	0.047	0.032	0.031	0.016	0.002	0.008	0.021	0.028	0.036
g__RB41	0.055	0.013	0.035	0.006	0.005	0.003	0.015	0.041	0.038	0.001	0.002	0.005
o__Rokubacteriales, g__ unassigned	0.029	0.055	0.023	0.005	0.003	0.001	0.017	0.035	0.023	0.001	0.001	0.001
g__Massilia	0.006	0.002	0.004	0.020	0.028	0.053	0.004	0.001	0.003	0.014	0.015	0.020
g__Bradyrhizobium	0.006	0.010	0.011	0.015	0.014	0.012	0.014	0.007	0.008	0.015	0.019	0.014
g__Streptomyces, g__ unassigned	0.008	0.011	0.009	0.021	0.016	0.018	0.010	0.006	0.008	0.010	0.012	0.015
f__Xanthobacteraceae, g__ unassigned	0.010	0.021	0.017	0.010	0.006	0.006	0.010	0.010	0.010	0.005	0.007	0.008
						D1						D2
o__Gaiellales, g__ unassigned	0.089	0.051	0.133	0.071	0.039	0.051	0.13	0.113	0.124	0.084	0.07	0.058
f__Nitrososphaeraceae, g__ unassigned	0.099	0.068	0.165	0.003	0.022	0.035	0.043	0.175	0.212	0.008	0.009	0.035
g__Sphingomonas	0.019	0.026	0.015	0.055	0.037	0.043	0.025	0.013	0.006	0.05	0.038	0.065
g__Arthrobacter	0.014	0.018	0.015	0.044	0.032	0.065	0.008	0.011	0.01	0.034	0.062	0.03
g__Candidatus_Udaeobacter	0.053	0.03	0.039	0.008	0.046	0.02	0.018	0.021	0.025	0.017	0.016	0.013
o__Vicinamibacterales, g__ unassigned	0.038	0.041	0.01	0.006	0.064	0.02	0.012	0.025	0.022	0.016	0.022	0.021
c__MB-A2-108, g__ unassigned	0.037	0.028	0.035	5E-04	0.01	0.004	0.014	0.033	0.069	0.003	0.002	0.008
f__Gemmatimonadaceae, g__ unassigned	0.03	0.022	0.016	0.016	0.019	0.015	0.035	0.041	0.022	0.015	0.027	0.019
g__Bacillus	0.032	0.019	0.014	0.055	0.032	0.022	0.023	0.02	0.007	0.024	0.039	0.027
c__KD4-96, g__ unassigned	0.034	0.033	0.031	0.002	0.023	0.021	0.016	0.022	0.021	0.01	0.01	0.015
f__SC-I-84, g__ unassigned	0.017	0.012	0.008	0.034	0.025	0.034	0.038	0.011	0.006	0.033	0.028	0.028
g__Gaiella	0.023	0.028	0.05	0.001	0.006	0.006	0.029	0.041	0.042	0.003	0.003	0.006
g__norank_f__67-14	0.018	0.027	0.031	0.017	0.011	0.023	0.025	0.029	0.031	0.014	0.021	0.01
g__Nocardioides	0.006	0.011	0.006	0.022	0.025	0.029	0.02	0.005	0.003	0.021	0.028	0.032
g__RB41	0.027	0.051	0.007	2E-04	0.014	0.002	0.011	0.019	0.018	0.002	0.001	0.005
o__Rokubacteriales, g__ unassigned	0.018	0.02	0.015	2E-04	0.004	9E-04	0.024	0.024	0.049	0.001	4E-04	0.006
g__Massilia	0.003	0.006	0.01	0.025	0.012	0.024	0.008	0.005	0.001	0.013	0.03	0.016
g__Bradyrhizobium	0.01	0.019	0.007	0.018	0.014	0.015	0.021	0.01	0.007	0.014	0.016	0.014
g__Streptomyces, g__ unassigned	0.007	0.013	0.008	0.018	0.015	0.014	0.014	0.01	0.006	0.018	0.019	0.013
f__Xanthobacteraceae, g__ unassigned	0.009	0.017	0.012	0.005	0.009	0.006	0.012	0.013	0.017	0.004	0.007	0.008

Table S6. Alpha diversity of each treatment group.

	CK					
Observed	3753	3100	3184	3663	3919	3676
Chao1	5362.636	4623.75	4710.782	5116.536	5175.287	4905
Shannon	6.217928	6.00889	6.078082	6.371886	6.489739	6.266427
Simpson	0.987857	0.991453	0.989035	0.992376	0.993517	0.987817
PD	237.7508	202.1566	203.7167	228.2513	262.6792	245.3979
	D0					
Observed	3806	3006	3437	3319	3851	3901
Chao1	5398.104	4318.181	5010.558	4599.526	5285.745	5365.209
Shannon	6.465992	5.78831	6.082505	6.392028	6.559208	6.627082
Simpson	0.993385	0.975921	0.983956	0.994125	0.994841	0.994562
PD	253.1475	195.2735	216.9152	219.7093	260.1052	260.7666
	D1					
Observed	4004	3838	3201	2997	4132	3722
Chao1	5742.328	5506.723	4675.434	4105.781	5541.748	5143.281
Shannon	6.450238	6.397714	5.853982	6.171404	6.73626	6.384187
Simpson	0.992355	0.994318	0.977437	0.992702	0.995901	0.992164
PD	245.9068	248.5008	215.1895	207.7436	262.5918	245.7474
	D2					
Observed	3784	3720	3012	3496	3431	3931
Chao1	5431.121	5412.537	4433.101	4786	4752.613	5473.165
Shannon	6.523632	6.108626	5.506628	6.428717	6.379018	6.592869
Simpson	0.995325	0.981459	0.964196	0.994587	0.993067	0.994433
PD	235.5145	234.6849	198.9558	243.7597	238.5957	266.2898

Table S7. Topological characteristics of empirically identified bacterial networks.

	CK	D0	D1	G3
Average degree	19.76	20.12	18.69	17.71
Average path length	3.34	4.26	4.92	4.39
Network diameter	4	10	13	11
Clustering coefficient	0.7	0.67	0.52	0.48
Heterogeneity	1.05	1.16	0.97	1.03
Centralization	0.04	0.06	0.04	0.04
Modularity	0.59	0.51	0.55	0.56

Table S8. Gene copy numbers related to soil nitrogen cycling.

	CK		D0		D1		D2		
	value	SD	value	SD	value	SD	value	SD	N
nagG	14698	1176	14698	2940	16034	3207	15520	3640	6
chiA	1735956	121517	1735956	260393	1893770	378754	1820000	396000	6
amoA2	820268	123040	820268	106635	894838	134226	901300	248000	6
nosZ1	5841012	845023	3841012	1152304	4190195	1257059	3420000	855000	6
nirS1	3689690	412144	1689690	160278	1897843	174849	1350000	477000	6
napA	2288464	640770	2288464	457693	2496506	499301	2620000	707000	6
gdhA	1072176.2	2037135	8765476.5	1608264	11696467	1754470	12360000	2960000	6
nifH	3520871	212922	1520871	456261	1659132	497740	1310000	196500	6

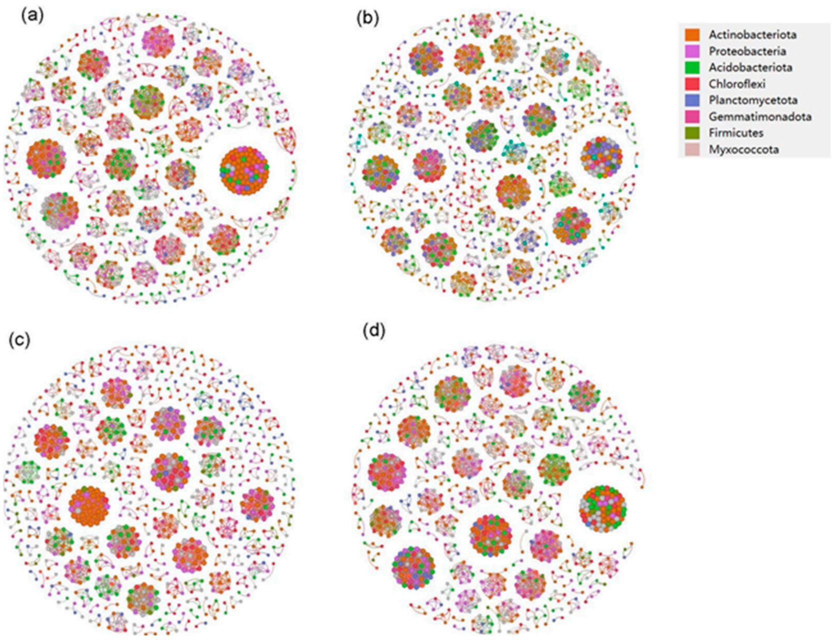

Figure S1. Bacterial co-occurrence networks across treatments: (**a**) Control (CK), (**b**) Treatment D0, (**c**) Treatment D1, (**d**) Treatment D2. Netwo rks were constructed using Spearman correlation ($|\varrho| > 0.6$, $p < 0.01$, FDR-corrected), where nodes represent amplicon sequence variants (ASVs) and edges represent significant co-occurrence relationships. Node colors indicate bacterial phylum-level taxonomy.

Table S9. Climate average for Fuxin City, Liaoning Province from June to October.

Month	Avg. Temperature (°C)	Avg. Precipitation (mm)
June	21–24	70–90
July	24–26	120–150
August	23–25	100–130
September	18–20	50–70
October	10–13	20–40

Table S10. Soil bulk density at 0–50 cm depth under each treatment.

	0–10 cm	10–20 cm	20–30 cm	30–40 cm	40–50 cm
CK	1.32±0.02b	1.51±0.06abc	1.55±0.08a	1.52±0.04a	1.51±0.02a
D0	1.55±0.05a	1.57±0.01a	1.55±0.04a	1.52±0.04a	1.50±0.02a
D1	1.32±0.03b	1.45±0.04c	1.48±0.03ab	1.52±0.01a	1.51±0.01a
D2	1.36±0.03b	1.44±0.08c	1.45±0.04b	1.43±0.04b	1.48±0.02a

Different lowercase letters indicate significant differences among different treatments ($p < 0.05$).

Table S11. Soil water content of 0-40cm in different growth periods.

	Jointing		Harvesting	
	0–20 cm	20–40 cm	0–20 cm	20–40 cm
CK	16.21±0.92cd	16.43±1.22cd	10.07±0.15f	9.45±0.62f
D0	15.53±1.40d	16.14±0.63d	10.21±0.08f	9.54±1.10ef
D1	17.59±0.59ab	16.72±1.00bcd	10.95±0.48cde	10.49±0.47de
D2	17.74±0.38ab	17.52±1.19abc	11.49±0.37bc	10.60±0.45d

Different lowercase letters indicate significant differences among different treatments ($p < 0.05$).
